# Mechanisms by stand density regulates soil multifunctionality via soil environment and microbial network topology in a *Pinus sylvestris* plantation

**DOI:** 10.3389/fmicb.2026.1796389

**Published:** 2026-04-08

**Authors:** Fengzi Li, Zhibo Wang, Yongning Hu, Xiuhua Wu, Lei Liu, Hongwei Yang, Ying Zhang, Yanqi Wang, Guangyu Hong

**Affiliations:** 1 Inner Mongolia Academy of Forestry Sciences, Hohhot, China; 2 Inner Mongolia Baotou Yellow River Wetland Ecosystem Observation and Research Station, Baotou, China; 3 Inner Mongolia Key Laboratory of Sandy Land (Desert) Ecosystem and Ecological Engineering, Hohhot, Inner Mongolia, China

**Keywords:** microbial community structure, microbial symbiotic network, Otingdag Sandy Land, soil microbial diversity, soil multifunctionality

## Abstract

In arid sandy plantations, stand density critically regulates belowground ecosystems, yet its effects on microbial network complexity, stability, and function are not fully understood. This study examined *Pinus sylvestris* var. *mongholica* plantations along a density gradient (Very high density (VHD): 2,450 trees ha^−1^, High density (HD): 1,633 trees ha^−1^, Moderate density (MD): 1,067 trees ha^−1^, Low density (LD): 583 trees ha^−1^) at two soil depths (0–20, 20–40 cm) in the Otingdag Sandy Land, integrating soil physicochemical, enzymatic, and microbial network analyses. Key findings were: (1) Soil organic carbon, total nitrogen, key enzyme activities [urease (URE), acid phosphatase (PHO), and nitrate reductase (NR)], and ecosystem multifunctionality showed a unimodal response, peaking at medium densities. (2) Microbial responses diverged: bacterial α-diversity changed but composition remained stable, whereas fungal composition was highly density-sensitive. Mid- to low densities promoted more complex, modular, and stable microbial networks. Mantel tests identified pH, URE, and Ammonium nitrogen (NH₄^+^-N, AN) as key drivers for bacterial phyla, and pH, Soil organic carbon (SOC), Total nitrogen (TN), PHO, and Polyphenol oxidase (PPO) for differentiating Ascomycota and Basidiomycota. (3) Random Forest regression identified microbial network stability as the top predictor of multifunctionality, surpassing diversity. Partial Least Squares Path Modeling (PLS-PM) analysis revealed that stand density enhances multifunctionality primarily by improving the soil environment, with microbial networks acting as environment-dependent regulators. This study demonstrates that moderate stand densities optimize microbial network resilience and ecosystem multifunctionality in sandy plantations, providing a novel perspective from microbial network stability.

## 1 Introduction

Afforestation is a core strategy for curbing land degradation and restoring ecosystem functions in arid and semi-arid regions (Rohatyn et al., 2022; Yang et al., 2023). The Otingdag Sandy Land serves as a crucial ecological barrier and dust source in northern China, making its vegetation restoration practices globally significant for regional ecological security. *Pinus sylvestris* var. *mongholica* Litv. (*Pinus sylvestris*), due to its exceptional drought resistance and sand-fixing capacity, has become a primary species for plantation establishment in this region (Zhu et al., 2025). However, large-scale monoculture management, while providing significant aboveground ecological benefits, also drives profound changes in belowground processes. Depletion of soil organic matter and nutrient pools, along with microbial functional imbalances, can potentially lead to soil fertility decline, threatening the long-term health and sustainability of plantation ecosystems (Yang et al., 2005; Zhang et al., 2022; Guo et al., 2025). Therefore, an urgent challenge is to simultaneously achieve aboveground vegetation restoration benefits and actively maintain or enhance the health, stability, and multifunctionality of the belowground ecosystem through precise management regulation.

As key drivers of Earth’s biogeochemical cycles, the structure, diversity, and interactions of soil microbial communities directly determine soil fertility, organic matter turnover, and ecosystem resilience (Zhu et al., 2022; Kong et al., 2024). Substantial evidence indicates that soil microbial biomass, activity, and enzyme activities related to carbon, nitrogen, and phosphorus transformations are tightly coupled with soil nutrient status, serving as highly sensitive biological indicators for assessing soil quality dynamics in plantations (Shi et al., 2025; Liu et al., 2023). Stand density, being the most direct and easily regulated key management target, reshapes the canopy structure, the quantity and chemical quality of litter input, root distribution patterns, and understory microclimate. These changes further influence the soil’s physical, chemical, and biological environment (Lu et al., 2025; Lei et al., 2021), thereby exerting a top-down filtering and restructuring effect on microbial communities. Preliminary studies suggest that the impact of stand density on soil microbes may be nonlinear. Medium density stands may be more conducive to soil organic matter accumulation and fungal diversity maintenance, whereas excessively high density may suppress bacterial diversity and simplify community structure (Yuan et al., 2021; Wang et al., 2023).

However, existing research has predominantly focused on descriptive characteristics of microbial communities (abundance, α-diversity). A significant knowledge gap remains concerning the systematic restructuring of microbial interspecific interactions triggered by density regulation and, more critically, how this restructuring subsequently influences macro-scale ecosystem properties, such as stability, resilience, and—importantly—multifunctionality. Ecosystem multifunctionality, which integrates multiple simultaneous ecosystem processes, provides a holistic measure of ecosystem service provision (Jobbágy and Jackson, 2001). Understanding whether and how management practices affect this integrated metric is essential for sustainability assessments. Crucially, the mechanisms linking management-induced shifts in microbial systems to changes in ecosystem multifunctionality remain poorly understood, particularly in the ecologically fragile Otingdag Sandy Land *Pinus sylvestris* plantations. A growing body of evidence demonstrates that forest management practices, including stand density regulation and thinning, can profoundly reshape the topological architecture of soil microbial co-occurrence networks (Lei et al., 2021; Gong et al., 2024). For instance, recent studies have shown that moderate thinning enhances network complexity and stability, thereby improving forest resilience to drought stress under global warming (Gong et al., 2024). Conversely, overly dense stands may lead to network simplification and increased fragility, potentially compromising ecosystem functions (Wang et al., 2023). The topological properties of these networks—such as complexity, modularity, and stability—have emerged as sensitive indicators of microbial community responses to environmental perturbations and management interventions, often outperforming traditional diversity metrics in predicting ecosystem multifunctionality (Yuan et al., 2021; Peng et al., 2024a). Advanced network inference methodologies, including those integrating metabolic complementarity principles, are now enabling a transition from descriptive correlation to mechanistic understanding of how management shapes microbial interactions and, consequently, ecosystem functional outcomes (Peng et al., 2024b; Wen et al., 2025).

This study aims to systematically unveil the comprehensive mechanisms by which stand density regulates belowground ecological processes in *Pinus sylvestris* plantations within the Otingdag Sandy Land, from a novel perspective of ecological complexity. We designed a continuous density gradient from very high to low (Very high density (VHD): 2,450 trees ha^−1^, High density (HD): 1,633 trees ha^−1^, Moderate density (MD): 1,067 trees ha^−1^, Low density (LD): 583 trees ha^−1^) as our research subjects. By integrating environmental factors such as soil physicochemical properties and enzyme activities, and employing high-throughput sequencing coupled with microbial co-occurrence network analysis, we endeavor to elucidate core questions at three levels. (1) How does stand density differentially shape the community diversity (alpha and beta diversity), composition, and key functional groups of soil bacteria and fungi? (2) How does density variation quantitatively reshape the topological structure of microbial co-occurrence networks and determine their stability? (3) How do these microbial network properties, particularly stability, relate to integrated soil ecosystem multifunctionality? Furthermore, what is the role of key soil environmental factors as drivers in the “density—environment—microbial network—ecosystem function” cascade? By quantitatively linking aboveground management to the complexity and stability of belowground microbial networks and their functional consequences, this study is expected to provide a solid scientific basis and a novel management perspective for the precise and sustainable silviculture of *Pinus sylvestris* plantations based on microbial ecological principles.

## 2 Materials and methods

### 2.1 Study sites and sample sites survey

The study area is located at the Duolun Otingdag Sandy Land Ecosystem National Positioning Observation and Research Station (115°51′–116°54′E, 41°46′–42°36′N) on the southern edge of the Otingdag Sandy Land, within Duolun County, Xilingol League, Inner Mongolia Autonomous Region. The region experiences a temperate continental climate transitioning from semi-arid to sub-humid and is also a transitional zone from the Yanshan Mountains to the Inner Mongolian Plateau. The multi-year average temperature is 1.6 °C with a short frost-free period (Li J. Z. et al., 2024). The mean annual precipitation is 385 mm, with 65–70% occurring from June to August. The area is characterized by strong winds year-round, with an average annual wind speed of 4.3–4.7 m s^−1^. Spring is particularly dry and windy, frequently featuring blowing sand and dust storm events (Qi et al., 2021). The predominant soil types are Chestnut soil and Aeolian sandy soil. Chestnut soil is mainly composed of fine sand with poor pedogenesis, low clay content, and a loose structure. Aeolian sandy soil remains in the parent material stage without distinct soil development features. Plantations in this area were primarily established in the late 20th century through national projects such as the Grain for Green Program and the Beijing-Tianjin Sand Source Control Program, with species including *Pinus sylvestris*, *Prunus sibirica* L., and *Ulmus pumila* L. The *Pinus sylvestris* plantations are over 20 years old, and the understory vegetation is dominated by shrubs such as *Salix gordejevii* Y. L. Chang & Skvortzov and *Caragana korshinskii* Kom., and herbs including *Leymus chinensis* (Trin. ex-Bunge) Tzvelev, *Agropyron cristatum* (L.) Gaertn., *Lespedeza bicolor* Turcz., and *Artemisia desertorum* Spreng (Li et al., 2017).

The experimental plots were established in the *Pinus sylvestris* plantation demonstration area, located in Duolun Nuor Town, Duolun County, Inner Mongolia, China. The plantation was established in 2011 using 3-year-old container-grown seedlings, with varying initial planting densities achieved through different row and column spacings. No thinning or other anthropogenic management interventions were conducted within the plots after afforestation. Soil survey was conducted in August 2025 across the different initial planting densities. Four distinct stand density levels were selected in areas with comparable site conditions for sampling, 583 trees ha^−1^, 1,067 trees ha^−1^, 1,633 trees ha^−1^, and 2,450 trees ha^−1^, representing the gradient as low density, moderate density, high density, and very high density (Table 1). For each stand density, three temporary standard sample plots, each measuring 30 m × 30 m, were established. All sample plots were spaced at least 50 m apart to ensure spatial independence. To ensure soil sample homogeneity, within each temporary standard sample plot, soil samples were collected from four directions (east, south, west, north) using a 5 cm diameter soil auger from the 0–40 cm layer (0–20 cm, 20–40 cm). Samples from each plot were thoroughly mixed to form two composite samples. In total, we obtained 48 samples from 6 replicates of 2 soil layers in 4 stand density plots. The composite samples were sieved through a 2 mm mesh to remove stones and plant debris. Approximately 1 kg of soil was placed into a sterile sealed bag and transported to the laboratory in a −20 °C vehicle freezer. A portion of each sample was air-dried indoors. Subsamples were then oven-dried at 105 °C for 6 h for the determination of pH, organic matter, and total nitrogen. Another portion was stored at 4 °C for subsequent analysis of readily oxidizable organic carbon, ammonium nitrogen, nitrate nitrogen, and enzyme activities. Separate, independent samples dedicated to microbial community analysis were preserved in liquid nitrogen during transport to maintain biological integrity. Upon arrival at the laboratory, these samples were immediately transferred to a −80 °C freezer for storage. To ensure analytical reliability, three replicate subsamples were prepared from each composite sample for subsequent soil microbial community analysis.

**Table 1:** Basic information of the survey sample site.

Treatment	Stand density	Longitude	Latitude	TH (m)	DBH (cm)	UBH (m)	CB (m)
LD	583 trees ha^−1^	116°27′56″E	42°13′17″N	6.40a	12.43b	0.43c	3.19b
MD	1,067 trees ha^−1^	116°45′13″E	42°11′50″N	5.88b	15.77a	1.37a	3.67a
HD	1,633 trees ha^−1^	116°27′46″E	42°13′23″N	5.19c	10.50b	1.07b	3.09b
VHD	2,450 trees ha^−1^	116°45′22″E	42°11′42″N	5.02c	8.65c	1.21b	2.84c

VHD, HD, MD, and LD represent extremely high density, high density, medium density, and low density, respectively.

### 2.2 Soil physicochemical property measurements

Soil chemical properties were assessed using various analytical techniques. Soil pH was measured in a 1.2.5 soil-to-water suspension after 30 min of extraction using a pH meter (Mettler Toledo, Shanghai, China). Soil water content was determined by the oven-drying method. Soil organic carbon (SOC) content was measured using the potassium dichromate heating method. Easily oxidizable organic carbon (OSOC) is typically determined using the potassium permanganate (KMnO₄) oxidation method. Total nitrogen (TN) was quantified by the Kjeldahl method. Total phosphorus (TP) was determined by the molybdenum-antimony colorimetric method after sodium hydroxide digestion. Total potassium (TK) was determined by flame photometry or atomic absorption spectroscopy after digesting the samples with hydrofluoric-perchloric acid or fusing with sodium hydroxide. Ammonium nitrogen (NH₄^+^-N, AN) and nitrate nitrogen (NO₃^−^-N, NN) was extracted with 1 mol/L potassium chloride solution from fresh soil samples and determined by continuous flow analyzer (SmartChem 200, AMS Alliance, Rome, Italy).

This study measured the activities of four hydrolases related to soil carbon, nitrogen, and phosphorus cycling. Polyphenol oxidase (PPO), urease (URE), acid phosphatase (PHO), and nitrate reductase (NR). All enzyme assays were performed using fresh soil samples. Each sample was analyzed in triplicate. Controls without soil and without substrate were included to correct for non-enzymatic reactions and soil background interference. Enzyme activities were measured using a 96-well multifunctional enzyme analyzer (SuPerMax 3100, Shanghai Shanpu Biotechnology Co., Ltd., Shanghai, China) following the instructions of Solarbio kits (Beijing Solarbio Science and Technology Co., Ltd., Beijing, China). URE activity was expressed as per μg NH₃^−^-N g^−1^ d^−1^, determined from the fluorescence absorption at 630 nm of a 0.25 g soil sample. PHO activity was expressed as per nmol phenol g^−1^ d^−1^, determined from the fluorescence absorption at 660 nm of a 0.25 g soil sample. PPO activity was expressed as mg purpurogallin g^−1^ d^−1^, determined from the fluorescence absorption at 430 nm of a 0.04 g soil sample. NR activity was expressed as μmol NO₂^−^ produced g^−1^ d^−1^, measured from the fluorescence absorption at 520 nm of a 0.05 g soil sample.

### 2.3 DNA extraction and high-throughput sequencing

After retrieval from storage, soil subsamples (0.2–0.5 g) were immediately placed in centrifuge tubes containing extraction lysis buffer and homogenized using a Tissuelyser-48 high-throughput tissue grinder (Shanghai Jingxin, Shanghai, China) at 60 Hz. Total microbial genomic DNA was extracted from 0.5 g of fresh soil using the MagBeads FastDNA Soil DNA Extraction Kit (116564384, MP Biomedicals, Irvine, CA, United States) following the manufacturer’s protocol. DNA quality and concentration were assessed by 1.0% agarose gel electrophoresis and a NanoDrop® ND-2000 spectrophotometer (Thermo Scientific, Waltham, MA, United States). Samples were then stored at −80 °C for subsequent analysis. The bacterial 16S rRNA gene and fungal ITS region was amplified using primer pairs 338F/806R (Johnson et al., 2019; Jackson et al., 2021) and ITS-1F/ITS-2R (Li M. et al., 2024), respectively, on an ABI GeneAmp® 9700 PCR thermal cycler (Foster City, CA, United States). Each 20 μL PCR reaction mixture contained. 4 μL of 5 × Fast Pfu buffer, 2 μL of 2.5 mM dNTPs, 0.8 μL of each primer (5 μM), 0.4 μL of Fast Pfu polymerase, and 10 ng of template DNA. The thermal cycling program included. Initial denaturation at 95 °C for 3 min. Twenty-seven cycles of denaturation at 95 °C for 30 s, annealing at 55 °C for 30 s, and extension at 72 °C for 45 s. final extension at 72 °C for 10 min. and hold at 4 °C. All amplifications were performed in triplicate. Amplicons were purified from 2% agarose gels using the AxyPrep DNA Gel Extraction Kit (Axygen Biosciences, Union City, CA, United States) and quantified using a Quantus™ Fluorometer (Promega, Madison, WI, United States). Purified amplicons were pooled in equimolar amounts and subjected to paired-end sequencing (2 × 300 bp) on an Illumina MiSeq PE300 platform (Illumina, San Diego, CA, United States) following standard protocols by Personal Biotechnology Co., Ltd. (Shanghai, China).

### 2.4 Data processing and analysis

Bioinformatic analysis of raw bacterial and fungal FASTQ sequencing data was performed using the QIIME2 pipeline (Bolyen et al., 2019). Quality filtering was conducted using fastp (v0.19.6) (Chen et al., 2018), followed by sequence merging using FLASH (v1.2.6) (Magoc and Salzberg, 2011). After quality control and merging, denoising of optimized sequences was performed using the DADA2 plugin (Callahan et al., 2016) within QIIME2 (Bolyen et al., 2019) with default parameters. Sequences resulting from DADA2 processing are referred to as amplicon sequence variants (ASVs). All sequences annotated as chloroplasts or mitochondria were removed. To minimize the influence of sequencing depth on subsequent alpha and beta diversity analyses, all samples were rarefied to 20,000 sequences per sample. After this normalization, the average sequencing coverage remained at 99.09%. Taxonomic annotation of ASVs was performed using a naïve Bayes classifier against the SILVA 16S rRNA gene database (v138) for bacteria and the UNITE database (Release 8.0^1^) for fungi.

SPSS Statistics (v25.0) was used for analyzing soil physicochemical properties, with one-way ANOVA and Tukey’s HSD test for multiple comparisons (*p* < 0.05). Alpha diversity indices (Chao1, ACE, Shannon, Simpson) were calculated to assess microbial richness and diversity. Beta diversity was examined via Principal Coordinates Analysis (PCoA) based on Bray–Curtis distances, with statistical testing using the Adonis function. Linear Discriminant Analysis Effect Size (LEfSe) was applied to identify differentially abundant taxa (LDA score > 4, *p* < 0.05) (Segata et al., 2011).

Bray-Curtis distances between microbial communities were calculated for Spearman correlation tests with environmental factor matrices. To reveal interactions among different soil microbial phyla across the four stands and soil layers, co-occurrence networks for bacterial and fungal communities were constructed. Topological parameters of each co-occurrence network were calculated, retaining species that appeared in at least 80% of all samples (He et al., 2017). Species-level analysis of microbial communities was performed using the Hmisc package (Chen et al., 2017) in R, and Spearman correlation coefficients were calculated. After generating matrices of correlation coefficients (r) and significance (*p*), data with |*r*| > 0.6 and *p* < 0.05 were imported into Gephi software (v0.10.1) to generate network graphs and calculate topological properties (Ren et al., 2022). Network stability was assessed by simulating random node removal (1–100% of nodes) using the fastnc software.^2^ The slope of the decline in natural connectivity after 1,000 averaged random simulations was calculated (Peng and Wu, 2016; Wu et al., 2021). Correlation analysis between microbial communities and environmental factors was performed following the method described by Zhang et al. (2016). Subsequently, Mantel tests using the vegan package (Guillot and Rousset, 2013) in R (v4.2.2) were conducted to analyze correlations between species-level abundances of bacteria and fungi and soil properties. Visualization was performed using the ggplot2 and corrplot packages.

To comprehensively assess soil multifunctionality, we selected seven key soil variables representing three core ecosystem processes based on their direct contributions to ecosystem functioning and their extensive application in soil quality assessments of plantation forests. These variables collectively capture the fundamental aspects of soil carbon, nitrogen, and phosphorus cycling, encompassing nutrient pool sizes (SOC, TN), available nutrient pools (AN, NN), and the key biochemical processes driving these cycles (URE, PHO, NR). This integrated selection enables a relatively comprehensive evaluation of the overall functional capacity of the soil ecosystem. For each soil sample, all seven variables were first normalized to a 0–1 scale using min-max normalization to eliminate dimensional differences among indicators. Subsequently, two complementary indices (M-index and T-index) are calculated for each soil sample. The M-index represents the average performance level of all evaluated functions for a given sample. It was calculated as the arithmetic mean of the seven normalized variables. The T-index assesses the breadth and synergy of multiple functions by counting how many functions are maintained at a high level simultaneously. Following established methods (Maestre et al., 2012), we calculated the T-index as follows. First, a threshold was set at 70% of the maximum observed value for each variable (Gamfeldt et al., 2008). For each variable, the maximum normalized value across all samples was calculated, and 70% of this maximum served as the cutoff. For each soil sample, we then counted the number of normalized variables that exceeded this 70% threshold. The T-index was calculated as the proportion of these high-performing functions. Together, these two complementary indices provide a robust and comprehensive assessment of the effects of stand density on soil ecosystem multifunctionality. Their responses to stand density, soil depth, and their interaction were analyzed using linear mixed-effects models. All analyses were conducted in R (v4.2.2) using the tidyverse, lme4, lmerTest, and emmeans packages. To identify the key predictors of multifunctionality, Random Forest regression was performed, incorporating microbial diversity, taxonomic composition, and network topological properties (Ren et al., 2025). The most important predictor was further examined via linear regression. Finally, Partial Least Squares Path Modeling (PLS-PM; plspm package) was applied to elucidate the direct and indirect pathways among stand density, soil environment, microbial networks, and multifunctionality. Model convergence was verified (achieved after 6 iterations), and path significance was assessed using 1,000 bootstrap resamples to calculate standard errors and 95% confidence intervals (Zhou et al., 2024).

## 3 Results

### 3.1 Effects of stand density on soil chemical properties across different depths

As shown in Table 2, across all stand densities, soil organic carbon (SOC), total nitrogen (TN), total phosphorus (TP), total potassium (TK), and nitrate nitrogen (NO₃^−^-N, NN) were generally higher in the surface layer (0–20 cm) than in the subsurface layer (20–40 cm). However, this vertical distribution pattern was less pronounced or even reversed under specific conditions. Notably, in the low-density (LD) stands, both SOC and NN exhibited comparable or slightly higher values in the subsurface layer compared to the surface layer. Soil pH shifted from slightly acidic to neutral with depth (*p* < 0.001). Across different stands, with increasing stand density, SOC, TN, ammonium nitrogen (NH_4_^+^-N, AN) (*p* < 0.05), and NN initially increased and then decreased (*p* < 0.001). The interaction between stand density and soil depth had a significant effect only on SOC and TN (*p* < 0.001). Surface soil (0–20 cm) exhibited significantly higher activities of polyphenol oxidase PPO, urease (URE), acid phosphatase (PHO), and nitrate reductase (NR) (*p* < 0.05) than subsurface soil (20–40 cm) (*p* < 0.001). Across different stands, URE, PHO, and NR activities initially increased and then decreased with increasing density (*p* < 0.001). The interaction between stand density and soil depth was significant only for URE and NR (*p* < 0.001).

**Table 2:** Characteristics of soil chemical properties and enzyme activities at different stand densities.

Stand density and soil layers	pH	SOC	EOC	TN	TP	TK	AN	NN	PPO	URE	PHO	NR
VHD-20	6.50 ± 0.04c	6.69 ± 0.26a	24.37 ± 3.67a	0.83 ± 0.02b	0.59 ± 0.01a	14.09 ± 0.27b	6.05 ± 0.29a	2.07 ± 0.23a	4.81 ± 1.89a	301.35 ± 9.56b	1.86 ± 0.32b	0.45 ± 0.05a
HD-20	6.64 ± 0.12c	10.16 ± 0.35a	24.86 ± 2.25a	1.25 ± 0.02a	0.64 ± 0.07a	13.94 ± 0.36b	6.11 ± 0.68a	2.67 ± 0.45a	2.32 ± 1.25b	423.48 ± 25.39a	2.90 ± 0.26a	0.69 ± 0.04a
MD-20	6.97 ± 0.06b	6.18 ± 0.33b	27.19 ± 2.31a	0.84 ± 0.04b	0.50 ± 0.04b	14.90 ± 0.15a	5.25 ± 0.91b	1.59 ± 0.26b	1.34 ± 0.66b	293.32 ± 21.14b	2.10 ± 0.51a	0.19 ± 0.16b
LD-20	6.91 ± 0.08b	2.99 ± 0.38c	19.43 ± 2.31a	0.56 ± 0.05c	0.44 ± 0.02c	14.77 ± 0.20a	3.70 ± 0.27c	1.82 ± 0.16b	1.74 ± 1.49b	295.59 ± 37.30b	1.35 ± 0.30b	0.28 ± 0.16b
VHD-40	7.02 ± 0.06a	6.37 ± 0.29b	24.65 ± 0.94a	0.81 ± 0.02b	0.53 ± 0.03b	13.51 ± 0.32c	4.37 ± 0.32c	1.45 ± 0.14b	3.96 ± 0.45a	251.15 ± 8.99c	1.49 ± 0.19b	0.10 ± 0.01c
HD-40	7.23 ± 0.11a	6.75 ± 0.26b	21.04 ± 1.27a	1.08 ± 0.03b	0.56 ± 0.04b	13.64 ± 0.32c	4.11 ± 0.94c	2.14 ± 0.33b	2.38 ± 0.84b	308.82 ± 7.91b	1.58 ± 0.10b	0.10 ± 0.01c
MD-40	7.19 ± 0.04a	3.19 ± 0.24c	23.21 ± 1.61a	0.68 ± 0.03c	0.46 ± 0.03c	14.59 ± 0.66a	3.26 ± 0.20c	0.85 ± 0.20c	0.88 ± 0.40c	236.07 ± 5.14c	0.88 ± 0.18c	0.10 ± 0.01c
LD-40	7.07 ± 0.03a	3.09 ± 0.42c	22.23 ± 1.61a	0.53 ± 0.01c	0.41 ± 0.02c	14.85 ± 0.28a	4.20 ± 0.83c	1.63 ± 0.19b	1.46 ± 0.60b	280.85 ± 4.68b	0.84 ± 0.06c	0.10 ± 0.01c
Forest type	***	***	ns	***	ns	ns	*	***	ns	***	***	***
Soil layers	***	***	ns	***	***	***	ns	***	***	***	***	*
SD × SL	ns	***	ns	***	ns	ns	ns	ns	ns	*	ns	*

Data for each condition are presented as mean ± SE. Means followed by the same letter within a column do not differ significantly by Tukey’s HSD test (*p* < 0.05, *N* = 6). Significant effect of two-way ANOVA: *p < 0.05, ***p < 0.001, ns, no significant effect. VHD, HD, MD, and LD represent extremely high density, high density, medium density, and low density, respectively; 20 and 40 represent the 0–20 cm and 20–40 cm soil layers, respectively.

### 3.2 Differences in soil microbial community composition

**Figure 1:**
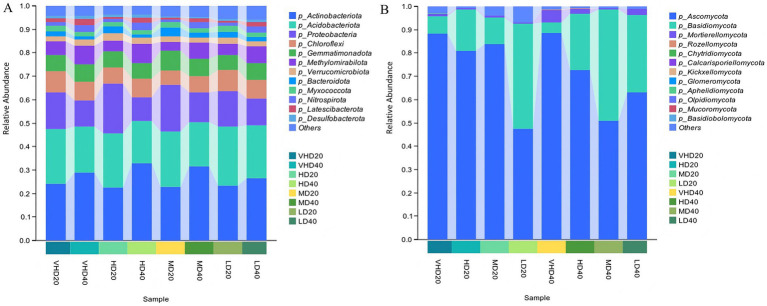
Proportion of the top 10 microorganisms in the relative abundance of bacterial communities **(A)** and fungal communities **(B)** in forest stands with different stand densities. VHD, HD, MD, and LD represent extremely high density, high density, medium density, and low density, respectively; 20 and 40 represent the 0–20 cm and 20–40 cm soil layers, respectively.

Subsequent analysis of 36 samples from different stand densities and soil layers using high-throughput sequencing identified 1,445 bacterial ASVs, spanning 41 phyla and 873 genera. It also identified 709 fungal ASVs, spanning 14 phyla and 498 genera. Figure 1A shows the relative abundances of the top ten bacterial phyla in different stands and soil layers. *Actinobacteriota* (26.65% ± 4.304%), *Acidobacteriota* (21.72% ± 2.617%), and *Proteobacteria* (14.7% ± 4.479%) were the dominant bacterial phyla across all four density stands (Figure 1A). Across different density stands, the proportions of *Actinobacteriota* and *Acidobacteriota* were higher in the subsurface soil than in the surface soil, whereas *Proteobacteria* showed a higher proportion in the surface soil. While the overall composition of the bacterial community was largely consistent across densities, the relative abundances of phyla varied. The relative abundances of *Actinobacteriota* and *Proteobacteria* initially increased and then decreased with decreasing stand density, peaking in HD (27.68, 16.36%). In contrast, *Acidobacteriota* and *Chloroflexi* initially decreased and then increased, reaching their highest proportions in LD (23.79 and 8.56%). *Ascomycota* (72.01% ± 20.52%) and *Basidiomycota* (23.89% ± 20.64%) were the dominant fungal phyla across all four density stands (Figure 1B). *Ascomycota* showed a higher proportion in surface soil across stands, while *Basidiomycota* was more abundant in subsurface soil. Fungal community composition was also broadly consistent, but phylum relative abundances varied. The relative abundance of *Ascomycota* gradually decreased with decreasing stand density, being highest in VHD (88.51%). Conversely, *Basidiomycota* gradually increased with decreasing density, reaching its highest proportion in LD (39.14%).

**Figure 2:**
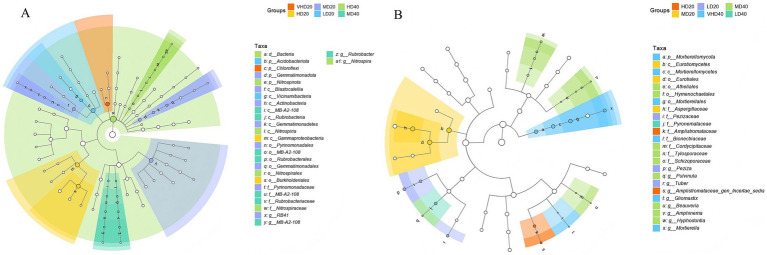
Lefse analysis was performed on soil bacterial **(A)** and fungal **(B)** communities in different soil layers of forest stands with varying stand densities. Significant taxonomic unit nodes are colored, and branch regions are shaded according to the highest-ranked group of the taxonomic unit. Nodes corresponding to taxonomic units without significant differential expression between sample groups are shown in white. Highly abundant and selective taxonomic units are marked. Abbreviations for different forest sites at various soil layer depths are provided in Figure 1.

As shown in Figure 2A, for the bacterial community, the biomarker significantly distinguishing VHD20 from other soil samples was *Chloroflexi*. Biomarkers in HD20 samples were primarily distributed within *Gammaproteobacteria* and *Burkholderiales*. MD20 sample biomarkers were mainly found in *Gemmatimonadota*, *Blastocatellia*, *Actinobacteria*, *Gammaproteobacteria*, *Pyrinomonadales*, *Gemmatimonadales*, and *Pyrinomonadaceae*. LD20 sample biomarkers were primarily *Acidobacteriota* and *Vicinamibacteria*. HD40 sample biomarkers were mainly *Nitrospirota*, *Nitrospinia*, *Nitrospirales*, *Nitrospiraceae*, and *Nitrospira*. MD40 sample biomarkers were predominantly *Rubrobacteria*, *Rubrobacterales*, *Rubrobacteriaceae*, and *Rubrobacter*. As shown in Figure 2B, for the fungal community, the biomarker significantly distinguishing HD20 from other samples was *Amplistromataceae* (wood-decaying fungi) and *Amplistromataceae genincertae sedis*. MD20 sample biomarkers were mainly *Eurotiomycetes*, *Eurotiales*, and *Aspergillaceae*. LD20 sample biomarkers were primarily *Pezizaceae* and *Tuber*. VHD40 sample biomarkers were mainly *Mortierellomycota*, *Mortierellomycetes*, *Mortierellales*, *Gliomastix*, and *Mortierella*. MD40 sample biomarkers included *Atheliales*, *Hymenochaetales*, *Cordycipitaceae*, *Tylosporaceae*, *Schizoporaceae*, *Pulvinula*, *Beauveria*, *Amphinema*, and *Hyphodontia*. LD40 sample biomarkers were primarily *Pyronemataceae*.

### 3.3 Differences in soil microbial diversity across stand densities

**Figure 3:**
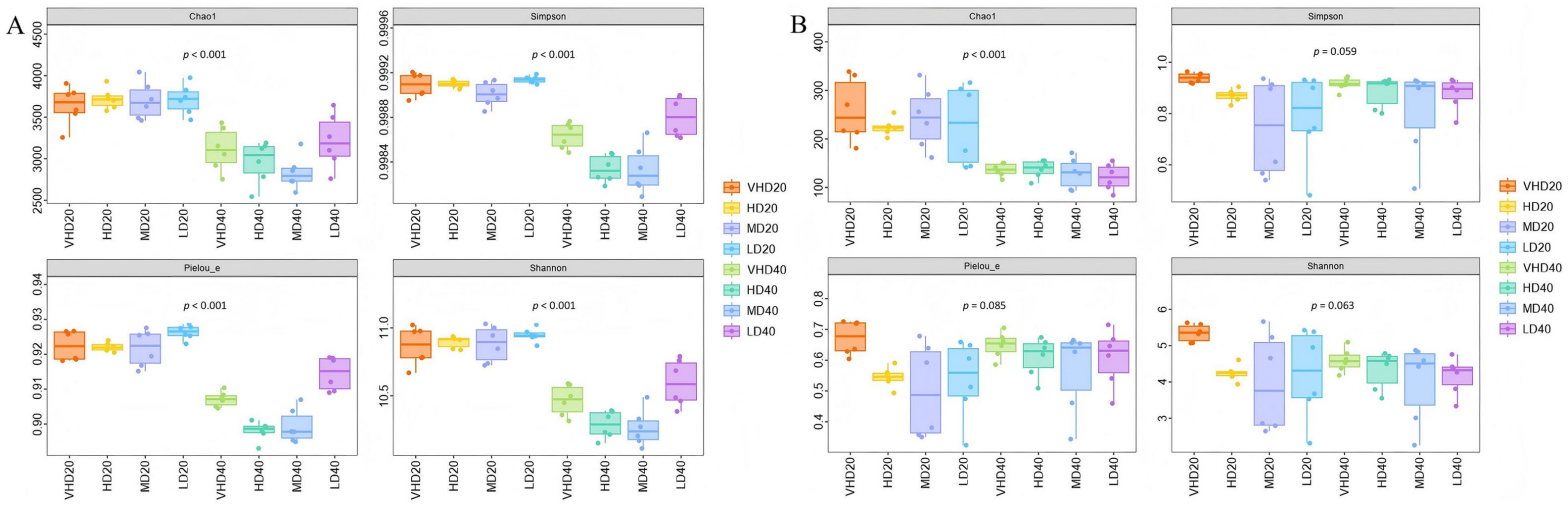
Alpha diversity of bacterial **(A)** and fungal **(B)** communities in different soil layers of different forests. Abbreviations for different forest sites at various soil layer depths are provided in Figure 1.

As shown in Figure 3A, significant differences (*p* < 0.05) were observed in bacterial Chao1, Simpson, Pielou’s evenness, and Shannon indices across densities. In surface soil, bacterial Chao1, Pielou’s evenness, and Shannon indices increased with decreasing stand density (*p* < 0.05), being highest in LD. The Simpson index initially decreased and then increased (*p* < 0.05), also highest in LD. In subsurface soil, all four bacterial indices initially increased and then decreased with decreasing density, though not significantly (*p* > 0.05). For fungi (Figure 3B), the Chao1 index differed significantly across densities (*p* < 0.05), while Simpson, Pielou’s evenness, and Shannon indices showed no significant differences. In surface soil, the fungal Chao1 index initially decreased and then increased with decreasing density (*p* < 0.05), peaking in MD. In subsurface soil, it initially increased and then decreased (*p* < 0.05), peaking in HD.

**Figure 4:**
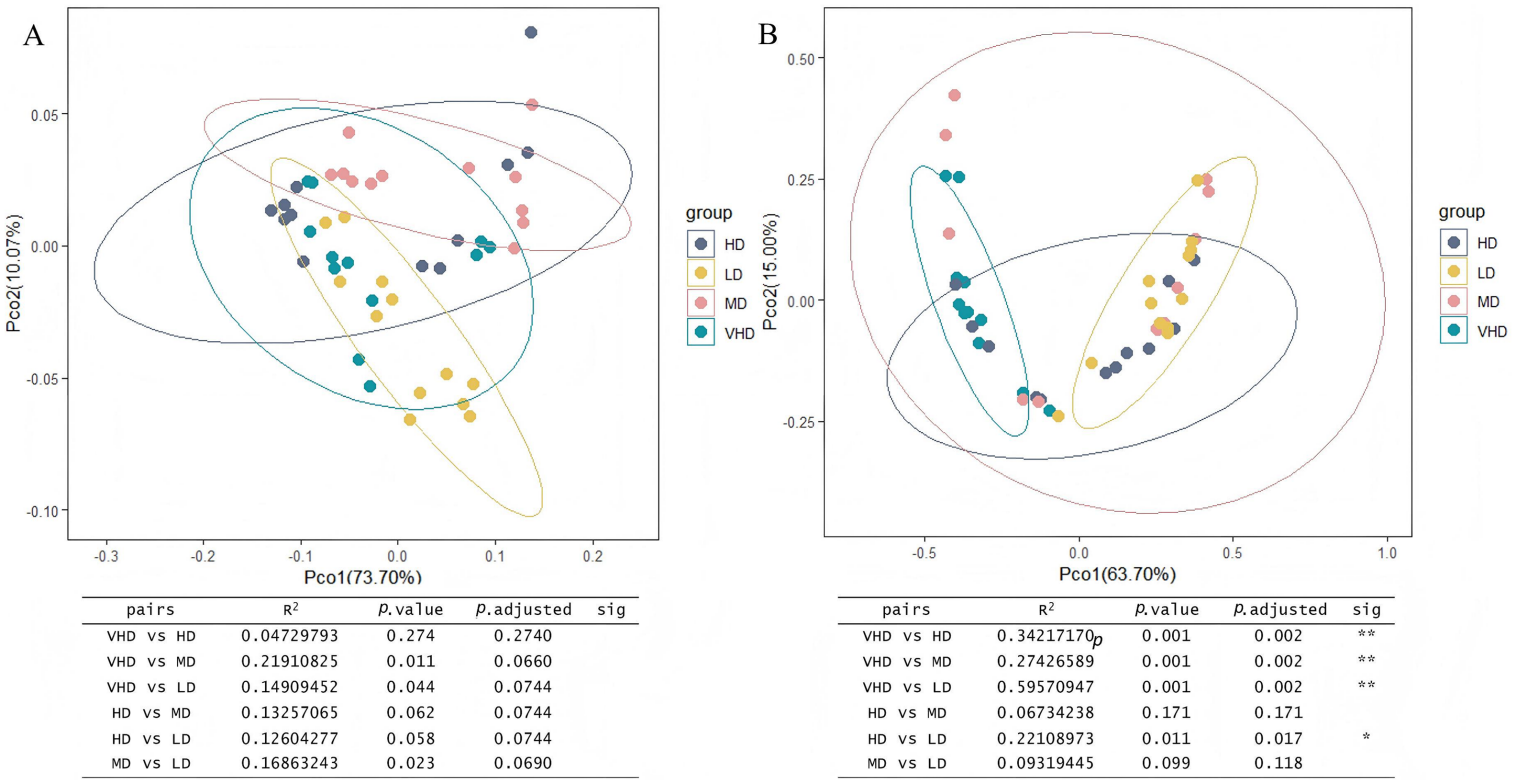
Principal coordinate analysis of different soil layers in different forest stands based on the Bray-Curtis distance. The beta diversity index was calculated by vegan package of R language, and Table **(A)** or **(B)** is the result of the multivariate analysis of variance performed using the Adon’s function, *p* < 0.05. Abbreviations for different forest sites at various soil layer depths are provided in Figure 1.

Principal Coordinates Analysis (PCoA) based on Bray-Curtis distances showed that for bacterial communities across stands and soil layers, the first two principal components explained 73.70 and 10.07% of the variation, respectively (Figure 4A). The confidence ellipses for the four stand densities largely overlapped, indicating no significant difference in bacterial community composition among forest types or soil depths (*p* > 0.05). For fungal communities (Figure 4B), the first two principal components explained 63.70 and 15.00% of the variation. Fungal community beta diversity differed significantly between VHD and HD/MD/LD, as well as between HD and LD (*p* < 0.05, Adonis test).

### 3.4 Microbial co-occurrence networks across stand densities and depths

**Figure 5:**
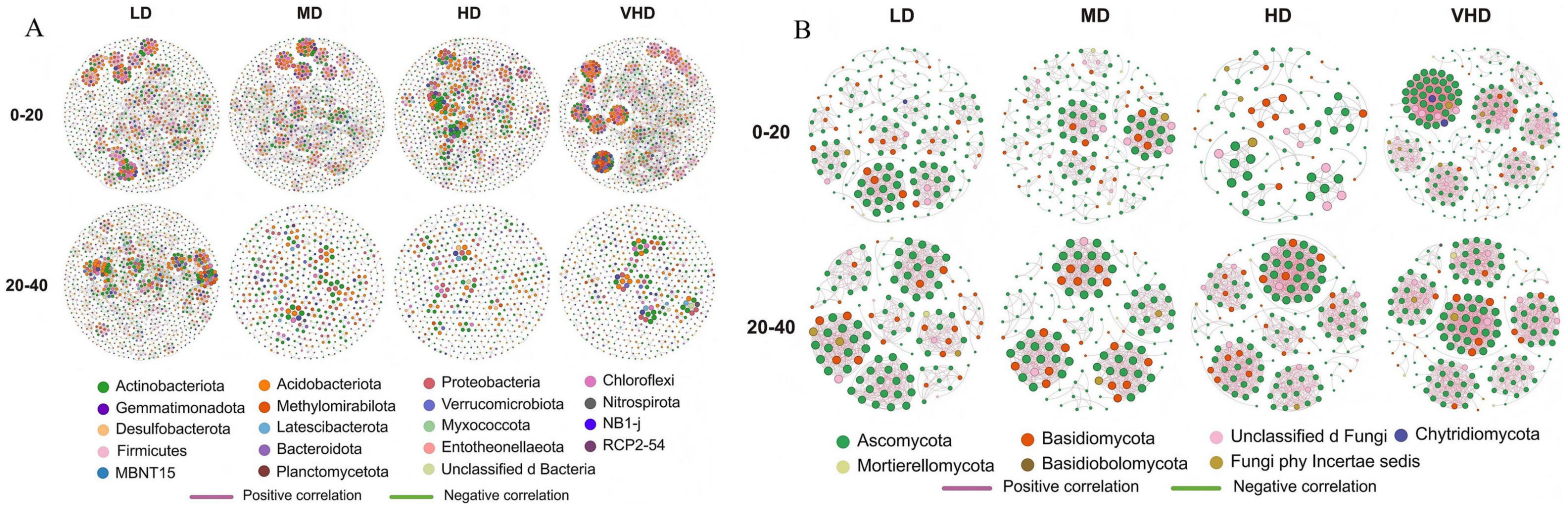
Correlation network of soil microbial bacterial **(A)** and fungal **(B)** communities in different stands and soil layers. Only the species association with extremely significant correlation was shown (|*r*| > 0.6 and *p* < 0.05), in which different nodes represent different species. The size of the node is proportional to the relative abundance of the species. Different colors represent the phylum of the species. The red connection indicates a positive correlation, the blue connection indicates a negative correlation, and the number of lines indicates the intensity of the connection between the nodes. Abbreviations for different forest sites at various soil layer depths are provided in Figure 1.

Nodes in the bacterial co-occurrence networks primarily belonged to 23 bacterial phyla. *Actinobacteriota*, *Acidobacteriota*, *Proteobacteria*, and *Chloroflexi* were the core phyla in the bacterial networks across densities (Figure 5). Positive correlations far outnumbered negative ones. In surface soil, VHD had the highest number of nodes, edges, and network density, while MD had the highest average path length, network diameter, clustering coefficient, and modularity. In subsurface soil, LD had the highest number of nodes, edges, average degree, and network density, but the lowest clustering coefficient and modularity. Bacterial networks showed relatively small differences in average path length and network diameter among density groups, indicating stable node connectivity efficiency. Nodes in the fungal co-occurrence networks primarily belonged to seven fungal phyla. *Ascomycota* and *Basidiomycota* were the core phyla (Figure 5). Compared to bacterial networks, fungal networks in surface soil exhibited lower modularity and average path length, with particularly evident in VHD (Table 3). VHD had the highest number of nodes, edges, and network density in both soil layers. Regarding network diameter, LD20 and MD40 were highest. For network density, VHD20 and HD40 were highest. Fungal networks showed relatively small differences in average path length and clustering coefficient among density groups, indicating stable node connectivity efficiency.

**Table 3:** Topological properties of bacterial co-occurrence network in different soil layers of four forests.

Community	Stand density	Nodes num	Edges num	Positive cor_num	Negative cor_num	Average degree	Average path length	Network diameter	Network density	Clustering coefficient	Modularity
Bacterial community	VHD20	924	5,569	4,607	962	12.05	7.30	24.64	0.01	0.88	0.88
	HD20	852	2,710	1,771	939	6.36	8.43	22.67	0.01	0.74	0.87
	MD20	905	3,875	3,002	873	8.56	8.60	24.64	0.01	0.84	0.91
	LD20	887	3,737	2,838	899	8.43	7.76	25.62	0.01	0.78	0.89
	VHD40	568	638	428	210	2.25	1.00	1.00	0.00	1.00	0.98
	HD40	543	487	331	156	1.79	1.00	1.00	0.00	1.00	0.99
	MD40	518	595	389	206	2.30	1.00	1.00	0.00	1.00	0.99
	LD40	918	3,362	2,180	1,182	7.32	9.80	32.52	0.01	0.74	0.85
Fungal community	VHD20	233	1,812	111	22	15.55	1.00	1.97	0.07	1.00	0.74
	HD20	98	133	589	12	2.71	1.03	1.97	0.03	0.98	0.91
	MD20	171	578	577	1	6.76	1.02	1.97	0.04	1.00	0.85
	LD20	173	601	1,807	5	6.95	1.01	2.94	0.04	1.00	0.87
	VHD40	173	1,438	1,191	2	16.62	1.00	1.96	0.10	1.00	0.81
	HD40	157	1,193	735	2	15.20	1.00	1.00	0.10	1.00	0.78
	MD40	129	649	648	1	10.06	1.00	1.97	0.08	1.00	0.78
	LD40	126	737	1,434	4	11.70	1.00	1.00	0.09	1.00	0.82

VHD, HD, MD, and LD represent extremely high density, high density, medium density, and low density, respectively; 20 and 40 represent the 0–20 cm and 20–40 cm soil layers, respectively.

**Figure 6:**
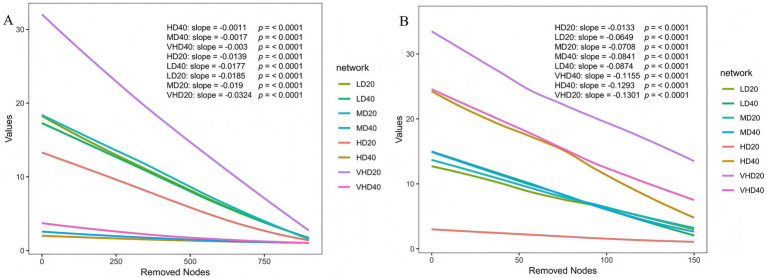
The natural connectivity of microbial networks in different soil layers of different stands. Natural connectivity of bacterial and fungal networks was calculated and evaluated by using fastnc (https://github.com/wgsst10Z/fastnc) software to reveal the stability of bacterial **(A)** and fungal **(B)** networks, respectively. Refer to Figure 1 for abbreviations of different forest stands in different soil depth.

Network stability, estimated by simulating node removal and measuring the rate of decline in natural connectivity (smaller absolute slope indicates greater stability), revealed fundamental differences between bacteria and fungi. For bacterial networks (Figure 6A), subsurface soil and HD exhibited higher stability, suggesting bacteria form more robust interactions under resource-limited or high-competition conditions. In contrast, fungal networks were more stable in surface soil and were relatively stable in HD and MD, while being most unstable in VHD (Figure 6B). This highlights the greater sensitivity of fungal communities, especially those involved in litter decomposition and mycorrhizal symbiosis, to changes in aboveground vegetation inputs compared to bacteria.

### 3.5 Relationship between soil microbial communities and soil chemical properties

**Figure 7:**
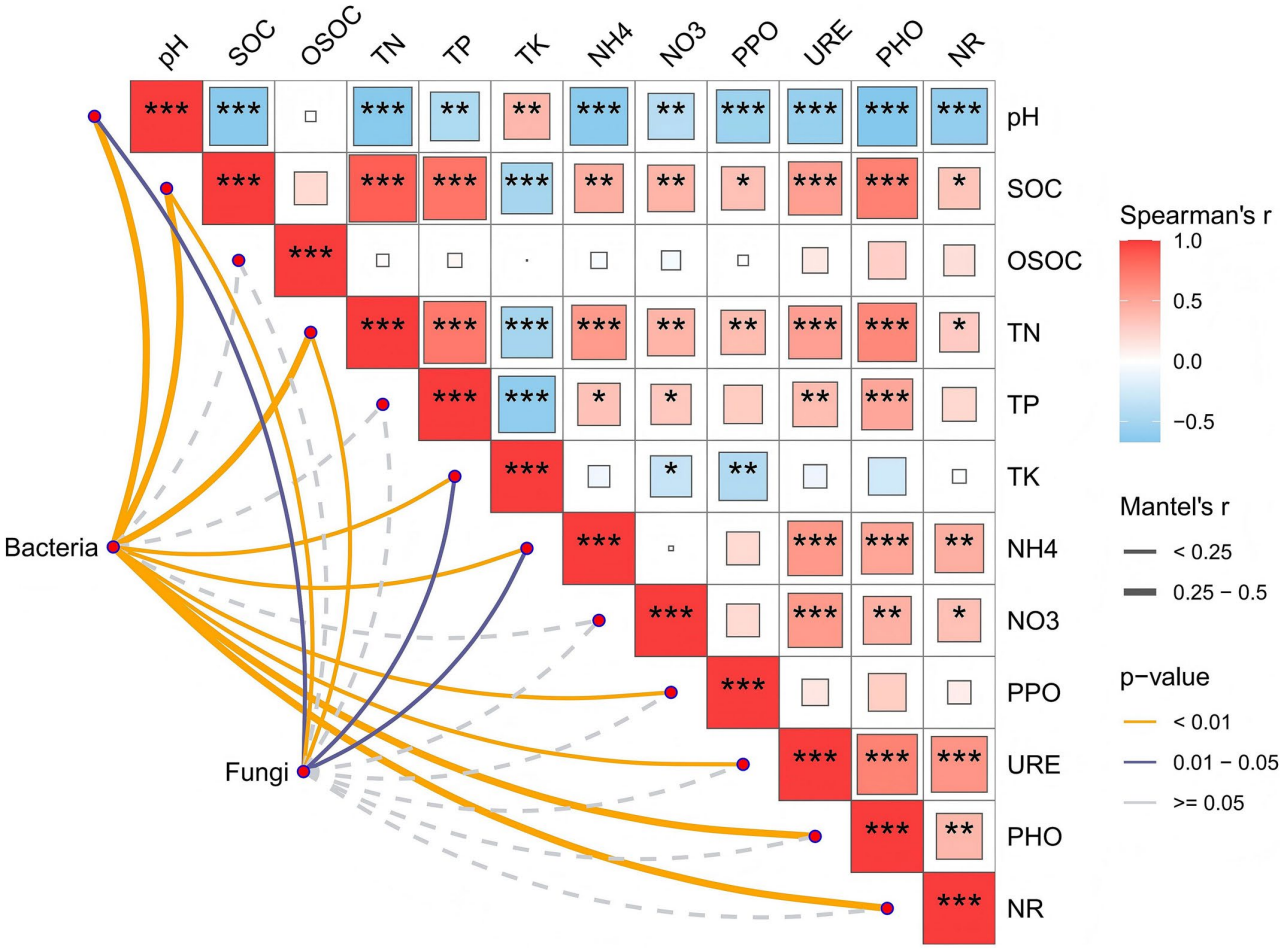
Mantel’s correlation analysis between soil properties and sol microbial communities. The color of the right box represents the spear-man correlation *r* value between environmental factors, *p < 0.05, **p < 0.01, ***p < 0.001, the left edge width corresponds to the Mantel’s *r* statistic of distance correlation, and the edge color represents the statistical significance *p* value based on 999 permutations.

**Figure 8:**
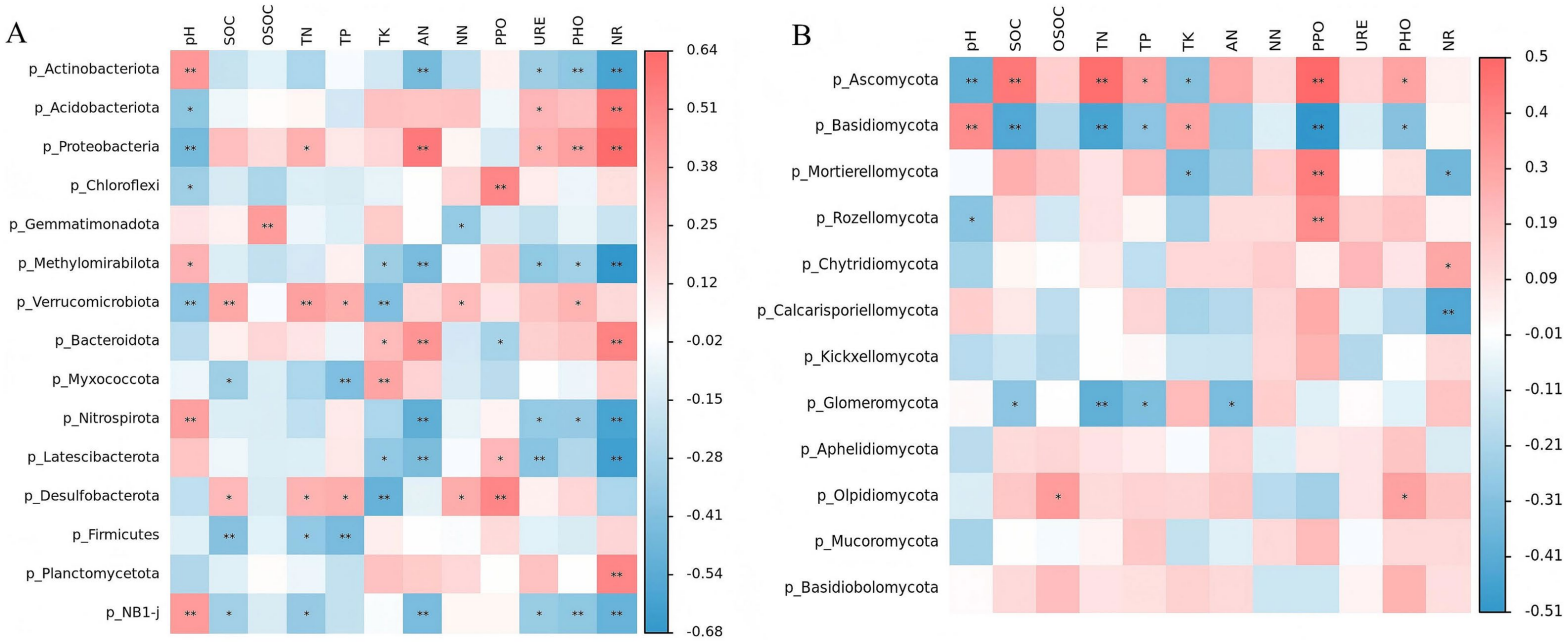
Correlations between the top 10 phyla in relative abundance and environmental factors in bacterial **(A)** and fungal **(B)** communities. The color represents the correlation coefficient, the black asterisk is the *p*-value of the correlation, the circle is the important value analyzed by multiple regression, and the bar chart on the right is the interpretation degree of environmental factors to each biological data in multiple regression.

In the bacterial community, significant positive correlations were observed between the bacterial community and pH, SOC, TN, TK, AN, as well as PPO, URE, PHO, and NR (*p <* 0.01; Figure 7). For the fungal community, in addition to significant positive correlations with SOC and TN (*p* < 0.01) and significant negative correlations with pH, TK, and AN (*p* < 0.05), its correlations with other soil physicochemical indicators were generally weak. At the phylum level for bacteria (Figure 8A), *Actinobacteriota* showed a significant positive correlation with pH and significant negative correlations with NR, PHO, URE, and AN. *Acidobacteriota* correlated positively with URE and AN and negatively with pH. *Proteobacteria* correlated positively with NR, PHO, URE, AN, and TN, and negatively with pH. Notably, OSOC had minimal influence on the bacterial community. For fungi (Figure 8B), *Ascomycota* correlated negatively with pH and TK, and positively with SOC, TN, TP, PPO, and PHO. *Basidiomycota* showed the opposite pattern, correlating positively with pH and TK, and negatively with SOC, TN, TP, PPO, and PHO. Notably, NO₃^−^-N and URE had minimal influence on the fungal community.

### 3.6 Responses of soil multifunctionality to stand density and relationships with microbial network characteristics

**Figure 9:**
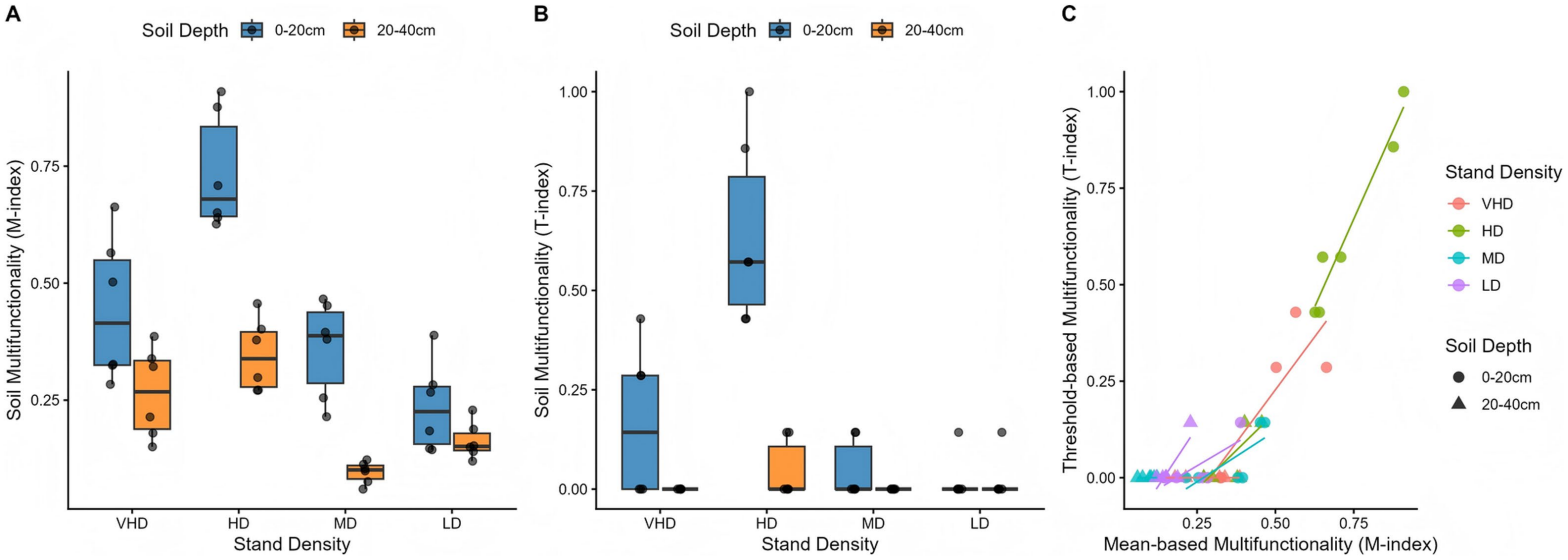
Response of soil multifunctionality to density gradient (**A**: M-index, **B**: T-index, **C**: Correlation between M-index and T-index). Refer to Figure 1 for abbreviations of different forest stands.

Based on key soil indicators, multifunctionality indices (M-index, T-index) were calculated. Both indices responded significantly to planting density and soil depth (Figure 9). In the 0–20 cm layer, the HD treatment showed the highest values (M-index: 0.80; T-index: 0.48–0.55). In the 20–40 cm layer, multifunctionality was lower overall, but the HD treatment still ranked highest (M-index: 0.42; T-index: 0.60–0.78), with its T-index exceeding its surface value. The response to density was nonlinear, with optimal function at HD and a decline at LD and VHD. In deeper soil, indices for MD and LD approached zero.

**Figure 10:**
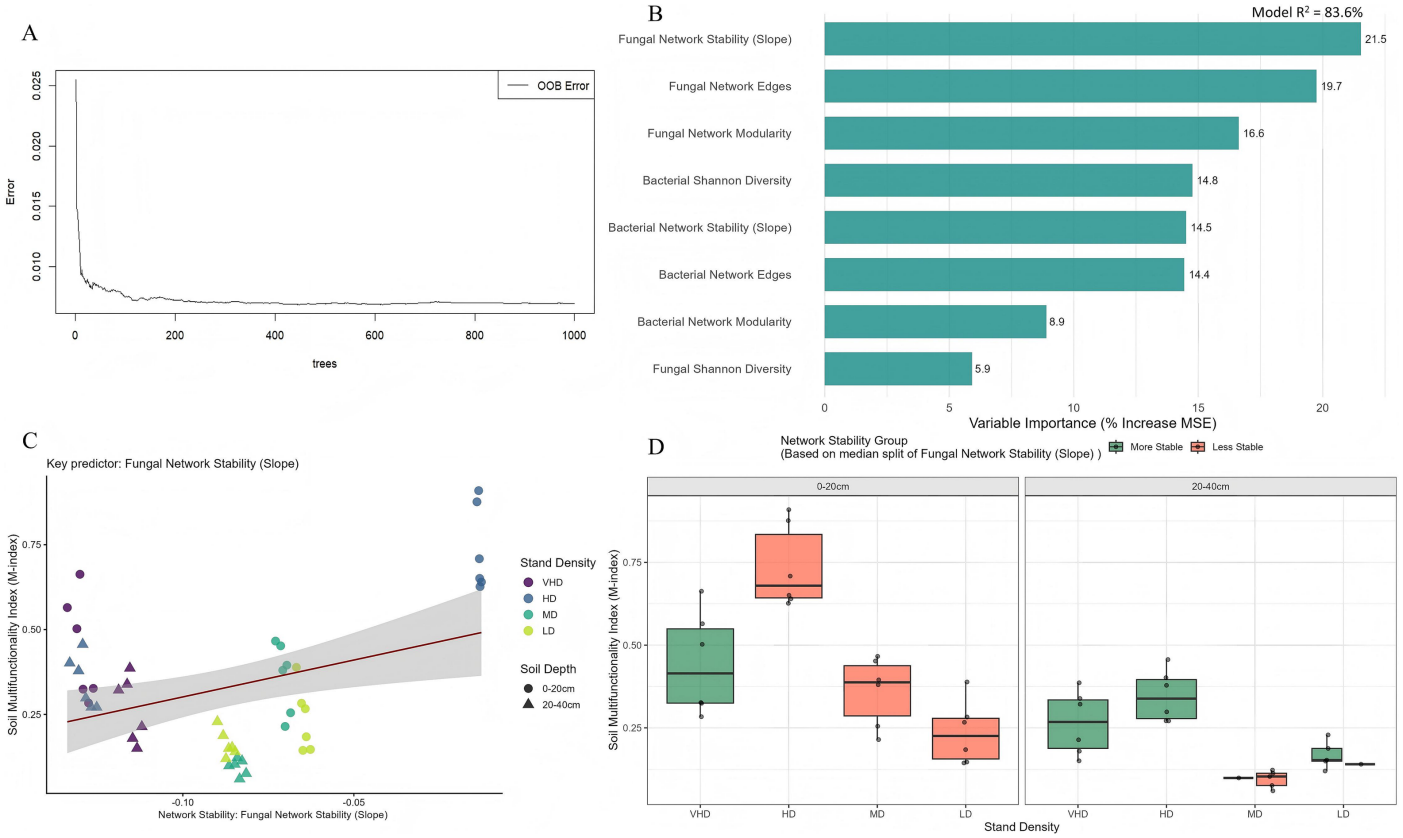
Ranking of microbial drivers for soil multifunctionality based on random forest variable regression analysis: **(A)** random forest model error versus number of decision trees, **(B)** key variables predicting soil multifunctionality, **(C)** relationship between network stability and soil multifunctionality, **(D)** response of multifunctionality to stand density across different network stability groups. Refer to Figure 1 for abbreviations of different forest stands.

The Random Forest model (*R*^2^ = 83.6%) identified microbial network properties as the top predictors of multifunctionality. Fungal network stability slope was most important (%IncMSE = 21.5%), followed by fungal network edges (19.7%) and modularity (16.6%) (Figures 10A,B). Fungal network stability was positively correlated with the M-index (Figure 10C). Grouping samples by stability median revealed an interaction with density: multifunctionality was higher and less variable in the high-stability group across all densities in the 0–20 cm layer, while the low-stability group showed lower and more variable function (Figure 10D). This pattern persisted in the 20–40 cm layer, where high stability buffered functional decline. In the low-stability group, multifunctionality did not improve even at MD/LD.

**Figure 11:**
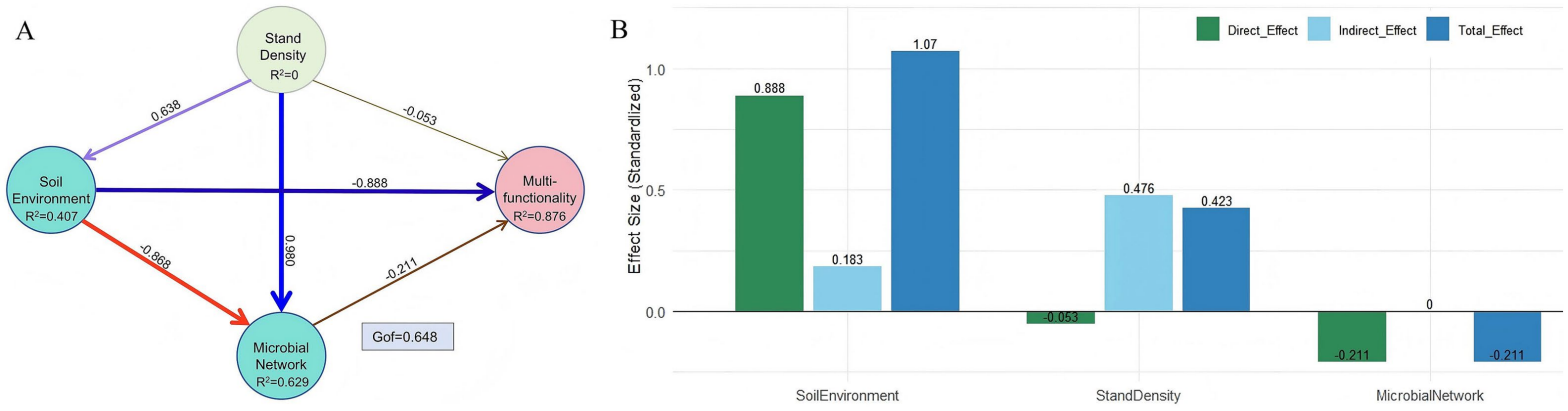
Partial least squares path model and effect decomposition for soil multifunctionality: **(A)** Schematic diagram of path analysis, **(B)** comparison of effects of various factors on soil multifunctionality. Refer to Figure 1 for abbreviations of different forest stands.

The PLS-PM showed good fit for endogenous variables (Soil Environment *R*^2^ = 0.407; Microbial Network *R*^2^ = 0.629; Multifunctionality *R*^2^ = 0.876; Figure 11A). Path coefficients indicated a strong direct effect of soil environment on multifunctionality (β = 0.888). Stand density positively affected the soil environment (β = 0.638) but had a weak direct effect on multifunctionality (β = −0.053). The microbial network had a direct negative effect (β = −0.211). The total effect of density on multifunctionality was 0.423, with 112.5% contributed by indirect effects (Figure 11B). The main indirect paths were “density–soil environment–multifunctionality” (effect = 0.566) and “density–microbial network–multifunctionality” (effect = −0.207). A chain mediation path “density–soil environment–microbial network–multifunctionality” had an indirect effect of 0.117.

## 4 Discussion

### 4.1 Response of soil chemical properties to stand density and soil depth

Soil nutrient contents (SOC, TN) exhibited a typical decreasing trend with soil depth, while pH increased. A key finding was the unimodal (increase-then-decrease) response of SOC, TN, AN, and NO₃^−^-N to stand density, indicating that mid- to low densities (MD/LD) were most conducive to soil nutrient accumulation and maintenance (Jobbágy and Jackson, 2001). When density was excessively high (VHD/HD), intense intraspecific competition suppressed tree growth, reduced litter quality, and led to excessive nutrient pumping by root systems, ultimately resulting in nutrient depletion. Interaction analysis further revealed that density and depth had a significant interactive effect only on SOC and TN. In the surface layer, density directly influenced these two primarily by regulating litter input; in deeper layers, its influence was indirect, mediated through changes in fine root distribution and leaching processes (Gao et al., 2025). Other indicators (TP, TK, pH) were predominantly controlled by regional factors such as soil parent material, thus showing only independent main effects of density and depth.

Soil enzyme activities were significantly higher in the surface layer, likely driven by the concentration of organic substrate inputs, microbial biomass, and favorable micro-environmental conditions there (Piotrowska-Długosz et al., 2021). URE, PHO, and NR activities also showed a unimodal pattern with density, indicating non-linear regulation of soil nutrient cycling by stand density. Mid- to low densities supported higher microbial activity and enzyme synthesis by optimizing litter input and the micro-environment, whereas excessively high density inhibited these metabolic processes due to intense competition and resource limitation (Huang et al., 2021). Interaction analysis showed that density and depth interacted significantly only for URE and NR. As central components of the nitrogen cycle, the depth-dependent response of URE (organic nitrogen mineralization) and NR (nitrate assimilation) to management practices hinges on the spatial distribution of nitrogen sources (litter-dependent in surface layers, root-turnover- and leaching-dependent in deeper layers) and the vertical heterogeneity of functional microbial communities (Hu C. C. et al., 2024; Liu et al., 2020). In contrast, PHO and PPO activities may be more uniformly regulated by overall soil phosphorus status or lignin-substrate abundance, hence the absence of a significant interaction.

### 4.2 Response mechanisms of microbial community abundance and diversity to stand density and soil depth

The response strategies of soil bacterial and fungal communities to the stand density gradient were fundamentally divergent. The shift in bacterial communities from oligotrophic (*Acidobacteriota*) to copiotrophic types (*Proteobacteria*) was closely associated with both soil depth and the density gradient, consistent with microbial energy limitation theory (Fierer et al., 2007). The abundance of copiotrophic taxa peaked under medium densities (HD/MD), likely due to an optimal balance of resource inputs and microenvironmental conditions (Cleveland and Liptzin, 2007). In contrast, the fungal community exhibited a clear functional transition: LD promoted an increase in ectomycorrhizal fungi (*Tuber*), enhancing symbiotic networks (Van der Heijden et al., 2008), while VHD enriched saprotrophic fungi (*Mortierella*), reinforcing decomposition functions (Tedersoo et al., 2014). This reflects a trade-off between fast-cycling and stable-storage nutrient acquisition strategies (Six et al., 2006).

The response patterns of bacterial and fungal alpha diversity to density and soil depth were markedly different. Bacterial diversity increased monotonically with decreasing density in surface soil but showed a nonlinear, initial increase followed by a decrease in subsurface soil, reflecting a trade-off between resource competition and microenvironmental conditions (Slessarev et al., 2016; Zhao et al., 2023; Zhang J. N. et al., 2025; Rumpel and Kögel-Knabner, 2011; Jiao et al., 2022; Shu et al., 2021). In contrast, fungal diversity (except for the Chao1 index) was largely insensitive to density changes, indicating greater structural stability, potentially due to stronger dependence on soil substrates (Li et al., 2020). Differences in fungal richness peaks across depths (MD/HD) suggest that community assembly mechanisms may vary with soil depth, necessitating further functional analysis.

Beta diversity analysis indicated that soil fungal communities were markedly more sensitive to stand density than bacterial communities in the *Pins sylvestris* plantation. Fungal composition differed significantly between VHD and other densities, and between HD and LD, whereas bacterial composition remained stable across treatments (Forrester, 2019). This divergence stems from their distinct ecological strategies. Bacteria, as r-strategists, respond quickly to labile resource pulses, but their overall community structure appears resilient to the broader physicochemical changes induced by density (Fierer et al., 2007). In contrast, fungi, as K-strategists, are tightly coupled to aboveground factors like canopy structure and litter quality, which density directly alters, leading to compositional shifts in functional groups such as saprotrophs and mycorrhizal fungi (Zhao et al., 2023). This reveals fundamentally different response strategies: fungi undergo functional reassembly, with composition (beta diversity) changing while local richness (alpha diversity) remains stable through species replacement (Liu et al., 2024). Conversely, bacteria exhibit numerical regulation, maintaining compositional stability while their local richness fluctuates with resource levels. Fungi thus act as sensitive indicators of system change, while bacteria function as broad responders to resource flux, together underpinning belowground ecosystem function (Sawada et al., 2021).

### 4.3 Differences in soil microbial community structure

Network analysis indicated that both bacterial and fungal networks in VHD stands exhibited the highest connectivity and network density. This reflects enhanced interspecific interactions among microbes to maintain function under intensified resource competition. However, this high connectivity may represent a strained collaborative state with potentially lower ecological stability. In contrast, network topological parameters under MD and LD, such as higher modularity and longer average path lengths, indicated stronger modular structure and more distinct functional partitioning within the communities. Higher modularity is often considered a buffer mechanism enhancing ecosystem resistance to disturbance, suggesting that low to medium density management may help cultivate more resilient soil microbial communities (Gong et al., 2024). The response patterns of bacterial and fungal networks to density differed markedly. Core bacterial groups (*Actinobacteriota*, *Acidobacteriota*) remained stable across treatments, and positive correlations dominated, suggesting bacterial communities tend toward broad cooperation to cope with environmental stress. In contrast, the core fungal groups (*Ascomycota* and *Basidiomycota*) and their connection patterns were more sensitive to density changes. Specifically, *Ascomycota* (often saprotrophic) dominated in VHD, while *Basidiomycota* (containing many ectomycorrhizal fungi) were more active in LD. This confirms that fungal communities respond more directly and strongly to perturbations driven by aboveground vegetation changes than bacterial communities (Jiao et al., 2021). Stand density, by altering resource input and the microenvironment, profoundly influences the complexity and stability of the belowground microbial network. The more modular and functionally differentiated microbial interaction networks formed under low to medium density management may provide a superior biological foundation for maintaining long-term soil ecosystem function and resisting disturbances.

By simulating node removal, this study assessed the stability of microbial co-occurrence networks, revealing fundamental differences in how bacterial and fungal networks respond to stand density and soil depth. In bacterial networks, subsurface soil and HD stands exhibited higher stability. This suggests that in resource-limited or high-competition environments, bacterial communities maintain function by forming more robust interaction networks. Conversely, fungal networks were more stable in surface soil and were relatively stable under HD and MD, while being most unstable under VHD. This highlights the far greater sensitivity of fungal communities, especially those involved in litter decomposition and mycorrhizal symbiosis, to changes in aboveground vegetation inputs compared to bacteria (Li Q. Y. et al., 2024). This difference in response patterns stems from their distinct ecological strategies. As rapid responders, bacterial network stability may be more associated with the homogeneity of soil physicochemical properties. The stable microenvironment created by high density may favor the construction of robust bacterial interaction networks. Fungi, as key decomposers and symbionts, have network stability tightly linked to the quality and continuity of fresh organic matter (surface litter) input (Kong et al., 2025). VHD may degrade litter quality and worsen the microenvironment, thereby disrupting fungal interspecific cooperation and leading to network fragility. MD may create an optimal balance of litter input and microenvironmental conditions, supporting more stable collaboration among fungal functional groups.

### 4.4 Chemical drivers and functional differentiation in soil microbial community

The bacterial community showed significant correlations with the vast majority of soil physicochemical properties (pH, nutrients, enzyme activities). This indicates that bacteria, as rapid responders, are extremely sensitive to multidimensional changes in the soil microenvironment. In contrast, the fungal community correlated mainly with soil nutrient (SOC, TN) and pH, showing weaker responses to available nitrogen (NO₃^−^-N) and some enzyme activities (URE). This supports the common view that fungal community assembly depends more on stable resource substrates (recalcitrant carbon and total nitro-gen pools) and is less sensitive to short-term, labile resource fluctuations (Liu et al., 2024). At the phylum level, the association patterns between key taxa and chemical factors revealed their ecological functions. Among bacteria, Proteobacteria showed significant positive correlations with various nutrients (TN, AN) and hydrolase activities (URE, PHO, NR), high-lighting their role as copiotrophic opportunists actively involved in C-N-P cycling (Wang et al., 2025). Conversely, *Acidobacteriota*’s negative correlation with pH and positive correlation with URE aligns with its oligotrophic, acid-preferring nature. *Actinobacteriota*’s positive correlation with pH and negative correlations with nitrogen-transforming enzymes suggest a competitive advantage in microenvironments with higher pH and relatively weaker nitrogen transformation. The minimal influence of readily OSOC on the bacterial community may indicate that bacteria in this system rely on a broader spectrum of carbon sources (Hu Y. et al., 2024). The two major fungal phyla exhibited nearly opposite correlation patterns. Ascomycota correlated positively with SOC, TN, and decomposition enzymes (PPO, PHO), strongly supporting its dominant role in litter degradation and saprotrophic nutrition. *Basidiomycota* (containing many ectomycorrhizal fungi) showed negative correlations with these factors and a positive correlation with pH. This may indicate its ecological niche is more oriented toward assisting host plant nutrient acquisition via mycorrhizal symbiosis in mineral soil layers rather than direct participation in surface organic matter decomposition (Zhang Y. et al., 2025). The seesaw dynamic between them visually demonstrates the shift in energy al-location within soil from organic matter decomposition to plant nutrient uptake. Forest stand density management alters soil chemical properties (pH, SOC, TN), thereby selectively enriching bacterial and fungal functional communities with specific ecological strategies. This transformation in microbial functional composition serves as the critical microbiological mechanism linking aboveground management practices with the under-ground nutrient cycling process.

### 4.5 Structure deciphering the mechanisms of stand density on soil multifunctionality

By integrating multifunctionality indices and path model analysis, this study systematically elucidated the nonlinear response characteristics and cascading mechanisms through which plantation density regulates soil multifunctionality. The results show that soil multifunctionality peaked at the medium-high density (HD), exhibiting a distinct unimodal response pattern. This aligns with the “Intermediate Disturbance Hypothesis” in ecology (Connell, 1978). Moderate-density planting likely creates an optimal balance between litter input and microenvironmental conditions, thereby promoting the synergistic enhancement of soil carbon, nitrogen, and phosphorus cycling functions. Notably, the T-index for the HD treatment in the deeper soil layer was even higher than that in the surface layer, indicating better vertical integration of soil functions at this density. This may result from an appropriate root distribution improving the physical structure and biological activity of the deeper soil (Jobbágy and Jackson, 2001). The functional decline associated with excessively low or high densities, particularly the near disappearance of function in the deeper soil under MD and LD treatments, suggests that improper density management may lead to profound functional degradation. This carries important implications for the sustainable management of plantation forests.

The PLS-PM further clarified the mechanistic pathways of density regulation from a process-based perspective. The soil environment exhibited the strongest direct effect on multifunctionality (β = 0.888), confirming the central role of soil physicochemical properties in driving ecosystem functioning. This finding is consistent with conclusions from global-scale studies highlighting the dominance of soil properties in ecosystem multifunctionality (Delgado-Baquerizo et al., 2016). The influence of plantation density on multifunctionality was primarily mediated through indirect pathways. Among these, the “density—soil environment—multifunctionality” path contributed the most (effect = 0.566), indicating that density management regulates functional integration mainly by altering key soil attributes. This provides direct evidence for optimizing soil fertility through rational stand density.

The regulatory role of the microbial network presented complexity. Its direct negative effect on multifunctionality (β = −0.211) might be related to functional redundancy or competition associated with high network complexity. Excessively complex interaction networks could reduce the efficiency of resource allocation, echoing the “complexity-stability” trade-off relationship observed in some studies (May, 1972). More notably, the significant negative influence of the soil environment on the microbial network (β = −0.868) reflects a trend toward simplification of microbial interactions under improved nutrient conditions. This may stem from reduced competition and decreased niche differentiation in resource-rich environments (Fierer et al., 2007). The positive indirect effect generated by the density through the “soil environment—microbial network—multifunctionality” chain pathway (0.117) reveals the trade-off between the soil environment and the microbial network and its ultimate impact on functional regulation. In summary, our study constructs a complete pathway of “aboveground density management—soil environment improvement—microbial network modulation—multifunctionality integration.” It identifies the soil environment as the key hub connecting aboveground management with belowground functioning, while the microbial network plays an environment-dependent regulatory role. These findings provide a theoretical basis for optimizing soil multifunctionality through precise density regulation and offer a new perspective for the coordinated “aboveground-belowground” management of plantation ecosystems.

## 5 Conclusion

This study elucidates the response and adaptation mechanisms of the soil ecosystem in *Pinus sylvestris* plantations to stand density gradients from the novel perspective of microbial interaction network stability. It confirms that mid- to low stand densities (approximately 1,067–1,633 trees ha^−1^) represent not only an optimal “management window” for enhancing soil nutrient accumulation and functional synergy but are also crucial for fostering robust belowground microbial interaction networks and improving system recovery capacity. The core mechanism lies in the fact that appropriate stand density indirectly drives the reorganization of microbial communities—centered on network stability—primarily by improving the physicochemical soil environment, which acts as a key regulatory hub, ultimately leading to the integration and enhancement of ecosystem multifunctionality. This provides critical mechanistic evidence from microbial ecology to support the “close-to-nature management” paradigm in arid-zone plantations, clearly indicating that modern forestry management must integrate aboveground density regulation with the cultivation of belowground network resilience to achieve long-term ecosystem stability and sustainability.

Future research should Integrate metagenomics and metabolomics to quantitatively identify the keystone microbial taxa and functional genes underpinning network stability, and to clarify the specific flow of carbon and nitrogen fluxes among network nodes. And employ long-term in-situ monitoring or microcosm experiments to directly verify the causal contribution of microbial network robustness to the recovery of specific ecological functions, particularly in response to disturbances such as drought and nutrient stress.

## Data Availability

The datasets presented in this study can be found in online repositories. The names of the repository/repositories and accession number(s) can be found in the article/Supplementary material.

## Footnotes

1 https://unite.ut.ee/ (accessed November 2, 2025).

2
https://github.com/wqssf102/fastnc
